# Clinical factors associated with functional outcomes in patients with single subcortical infarction with neurological deterioration

**DOI:** 10.3389/fneur.2023.1129503

**Published:** 2023-03-23

**Authors:** Yi Yang, Yue He, Wei Han, JianHui Xu, ZhiRong Cai, Tian Zhao, YuanWei Shao, Ming Yu

**Affiliations:** ^1^Department of Neurology, Affiliated Hospital of Jiangsu University, Zhenjiang, China; ^2^Department of Radiology, Affiliated Hospital of Jiangsu University, Zhenjiang, China

**Keywords:** parent artery disease, single subcortical infarction, neurologic deterioration, 90-day functional outcome, ischemic stroke

## Abstract

**Objective:**

Factors that predict poor outcomes in patients with single subcortical infarction (SSI) may differ from those that predict poor outcomes in the SSI subgroup with neurological deterioration (ND). This study aimed to investigate the effect of ND on functional outcomes in patients with SSI and the clinical factors that predict poor outcomes in patients with SSI with ND (SSI-ND) and in all patients with SSI.

**Methods:**

Patients with SSI were consecutively enrolled in this study. ND was defined as an increase of ≥2 points in the National Institutes of Health Stroke Scale (NIHSS) total score, an increase of ≥1 point in the NIHSS subscore of consciousness or motor function, or any new neurological deficit.

**Results:**

A total of 255 patients were enrolled, and nine (3.53%) were lost to a follow-up. ND [adjusted relative risk (aRR) = 1.37, 95% confidence interval (CI) = 1.22–1.55, *p* < 0.001], female sex (aRR = 1.13, 95% CI = 1.03–1.24, *p* = 0.12), initial NIHSS (aRR = 1.08, 95% CI = 1.07–1.10, *p* < 0.001), and parental arterial disease (PAD) (aRR = 1.16, 95% CI = 1.07–1.26, *p* = 0.038) were associated with a poor 90-day outcome (the modified Rankin scale (mRS) > 2 points) in patients with SSI. In the SSI-ND subgroup, PAD (aRR = 2.15, 95% CI = 1.20–3.86, *p* = 0.01), glycosylated hemoglobin (aRR = 1.17, 95% CI = 1.01–1.35, *p* = 0.035), and severe NIHSS (aRR = 1.15, 95% CI = 1.06–1.25, *p* = 0.001) were predictive of a poor outcome, and PAD (aRR = 1.87, 95% CI = 1.19–2.95, *p* = 0.007) was correlated with higher/worsened NIHSS [> 2 points (median)]. For predicting poor outcomes in patients with SSI-ND with PAD, a more severe NIHSS (aRR = 1.09, 95% CI = 1.02–1.17, *p* = 0.01) was the only determinant, with a cutoff of 4.5 points, a sensitivity of 94.0%, and a specificity of 83.3%.

**Conclusions:**

ND is an independent predictor of poor outcomes in patients with SSI, and poor outcome determinants in the SSI-ND subgroup and in all patients with SSI are not identical. For patients with SSI-ND, PAD could aggravate ND and was therefore an essential predictor of poor outcomes.

## Introduction

The pathogenic mechanism of single subcortical infarction (SSI) involves the blockage of the orifice of the perforating artery by an atheroma in the parent artery or lipohyalinosis or fibrinoid degeneration of the perforating artery (1, 2). This mechanism differs from the pathogenic mechanisms of other stroke subtypes, such as large-artery atherosclerosis or small-vessel occlusion stroke. According to a recent study, there are numerous clinical factors that predict functional outcomes in patients with stroke due to different pathogenic mechanisms (3). In addition, several studies have demonstrated that the positional relationship between the infarct and the parent artery is a unique predictor of functional outcomes in patients with SSI (4, 5). Therefore, factors associated with functional outcomes in patients with SSI are different from those associated with other stroke subtypes.

Patients with SSI were more susceptible to neurological deterioration (ND) than those with other stroke subtypes (6–9). ND has been shown to be detrimental to the outcome of patients with stroke (10–12). However, it was demonstrated in studies that enrolled patients with stroke of all subtypes rather than simply focusing on the subpopulation of patients with SSI. It is still unknown whether ND has a definite effect on functional outcomes in patients with SSI. We speculated that patients with SSI with ND (SSI-ND) would have a lower poststroke quality of life than the general SSI population. Therefore, clinical factors that predict a poor functional outcome in the SSI-ND subgroup may differ from those established for the entire SSI population (13–16). This study aimed to investigate (1) the effect of ND on functional outcomes in patients with SSI and (2) clinical factors that predict a poor functional outcome in patients with SSI and SSI-ND to allow for early identification and management of patients with SSI-ND who are at a high risk of achieving poor outcomes.

## Materials and methods

### Study population

This study was conducted in accordance with the principles set forth in the Declaration of Helsinki (1964). Patients who were admitted to the stroke unit of the Affiliated Hospital of Jiangsu University between 1 August 2021 and 31 July 2022 and were diagnosed with SSI were consecutively enrolled. The inclusion criteria were as follows: (1) age ≥ 18 years and (2) admission within 7 days of symptom onset. There were several exclusion criteria. We first proposed a study to investigate the predictive value of parental arterial disease (PAD) for functional outcomes. However, as previously reported, stenosis of ≥ 50% in the extracranial artery ipsilateral to the infarct might affect the relationship between PAD and functional outcomes; thus, patients with that condition were excluded from our study (15, 17). Among patients with SSI in the territory of the middle cerebral artery, those with a stenosis of ≥ 50% on the ipsilateral extracranial carotid artery were excluded; among patients with posterior SSI, those with a stenosis of ≥ 50% on the ipsilateral extracranial vertebral artery were excluded. Other exclusion criteria included: (1) vascular disease attributed to non-atherosclerosis, such as dissection, vasculitis, Moyamoya disease, and similar conditions; (2) potential sources of cardioembolism, such as atrial fibrillation, myocardial infarction within 4 weeks of admission, cardiomyopathy, and valvular heart disease; (3) organ failure or malignant tumor; and (4) the modified Rankin scale (mRS) of > 2 points before the index stroke. This study was approved by the Medical Research Ethics Committee of the Affiliated Hospital of Jiangsu University. All participants gave their written consent and agreed to the publication of neuroimaging images without personal information.

### Baseline characteristics

According to the WHO criteria, acute ischemic stroke was diagnosed when there was acute neurological deficit that persisted for more than 24 h (18). On admission, clinical baseline characteristics, including sex, age, a history of hypertension, a history of diabetes mellitus, smoking, alcohol consumption, and previous stroke (ischemic or hemorrhagic stroke), of each patient were recorded. After admission, a neurologist assessed the severity of the neurological deficits in each patient according to the National Institutes of Health Stroke Scale (NIHSS), which was recorded as the patient's initial NIHSS. During hospitalization, all patients underwent neuroimaging, laboratory tests, dynamic electrocardiography, and echocardiography. All patients received medical treatment both during hospitalization and after discharge, in accordance with the guidelines for the treatment and secondary prevention of acute ischemic stroke (19).

### Definition of ND

Neurological deterioration was defined as an increase of ≥ 2 points in the NIHSS total score, an increase of ≥ 1 point in the NIHSS subscore 1a, 1b, or 1c (consciousness level), an increase of ≥ 1 point in the NIHSS subscore 5a, 5b, 6a, or 6b (motor), or any new neurological deficit (10). Symptoms of worsened or new neurological deficits should persist for ≥ 24 h. Early ND (END) was defined as ND occurring within 72 h of admission (20).

When the participant experienced ND, the NIHSS score of the participant was evaluated immediately and every 24 h until discharge. The most severe NIHSS was the score that reflected the most severe neurological deficit of each participant; a worsened NIHSS was defined as the patient's most severe NIHSS score minus his or her initial NIHSS score.

### Evaluation of SSI using cranial magnetic resonance imaging

**Figure 1:**
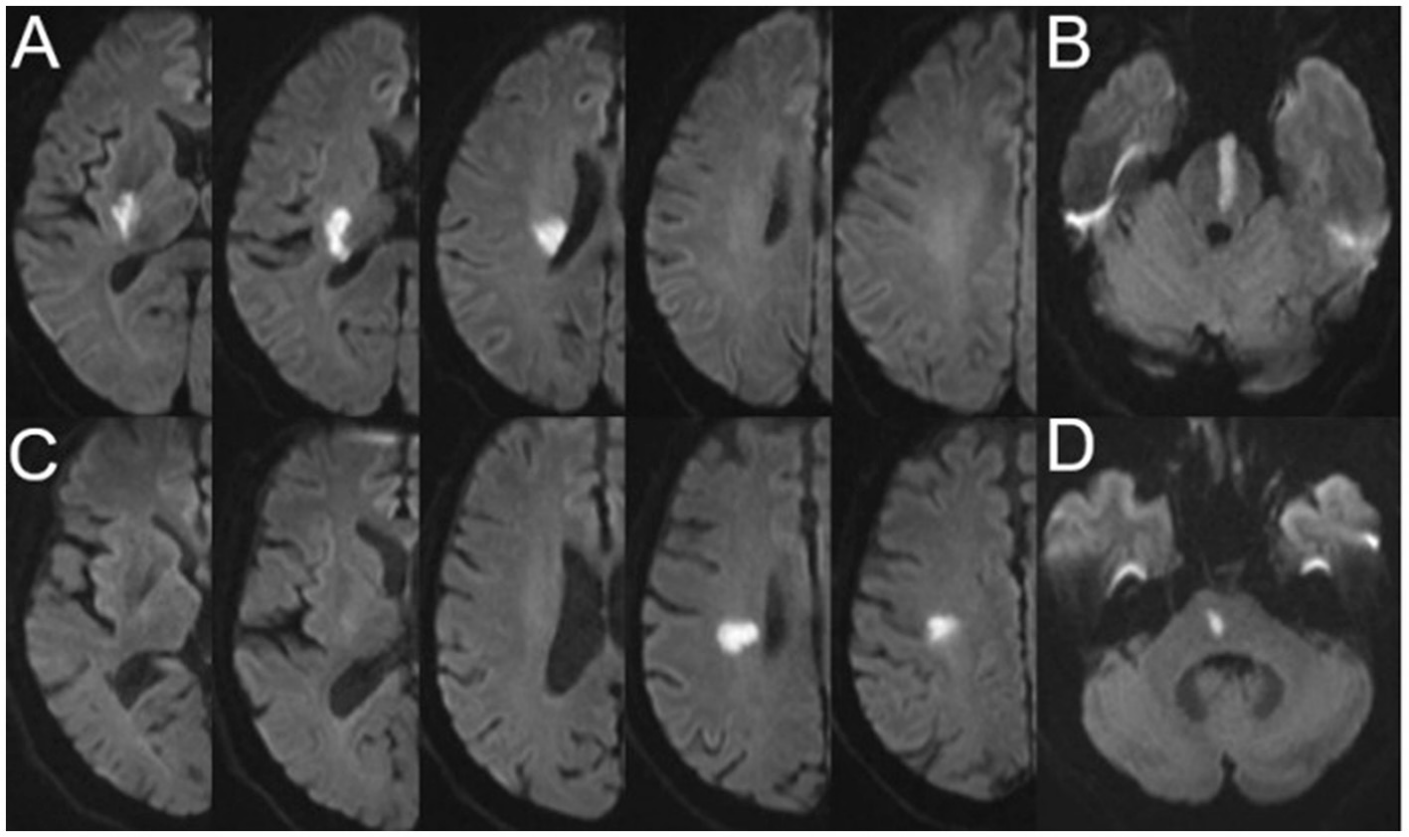
Representative cases of pSSI and dSSI. **(A)** pSSI in the anterior circulation; **(B)** pSSI in the posterior circulation; **(C)** dSSI in the anterior circulation; and **(D)** dSSI in the posterior circulation. pSSI, proximal single subcortical infarction; dSSI, distal single subcortical infarction.

The characteristics of SSI were evaluated using a 3.0T superconducting nuclear magnetic resonance apparatus (Siemens, Germany). Imaging sequences included T1-weighted imaging (T1WI), T2-weighted imaging (T2WI), fluid-attenuated inversion-recovery (FLAIR), and diffusion-weighted imaging (DWI) (slice thickness: 0.8 mm; interval: 0 mm; field of view: 230 mm × 230 mm). The most common parameters used in the imaging sequences were as follows: T1WI: repetition time (TR) = 450 ms, echo time (TE) = 10 ms; T2WI: TR = 4,350 ms, TE = 95 ms; FLAIR: TR = 8,200 ms, TE = 113 ms; and DWI: TR = 4,000 ms, TE = 97 ms. The presence of a single perforating infarction detected by DWI in the supply area of the middle cerebral, vertebral, or basilar artery is referred to as SSI (21). A perforating infarct in the middle cerebral artery was defined as an infarct detected in the supply area of the lenticulostriate arteries, including the basal ganglia (globus pallidus, putamen, thalamus, and caudate), corona radiata, and internal capsule (22). A perforating infarct in the basilar artery was defined as an infarct limited to the paramedian pontine area and in the vertebral artery was defined as an infarct limited to the medial medulla (16). According to previous studies and the positional relationship between the infarct and the parent artery, we classified SSI into proximal SSI (pSSI) and distal SSI (dSSI) (4, 21). pSSI was defined as an infarct that was located adjacent to the parent artery, extending toward the basal surface of the parent artery, whereas dSSI was defined as an infarct that existed only in the distal area of the parent artery (9, 23) (Figure 1).

### Evaluation of intracranial and extracranial atherosclerosis

**Figure 2:**
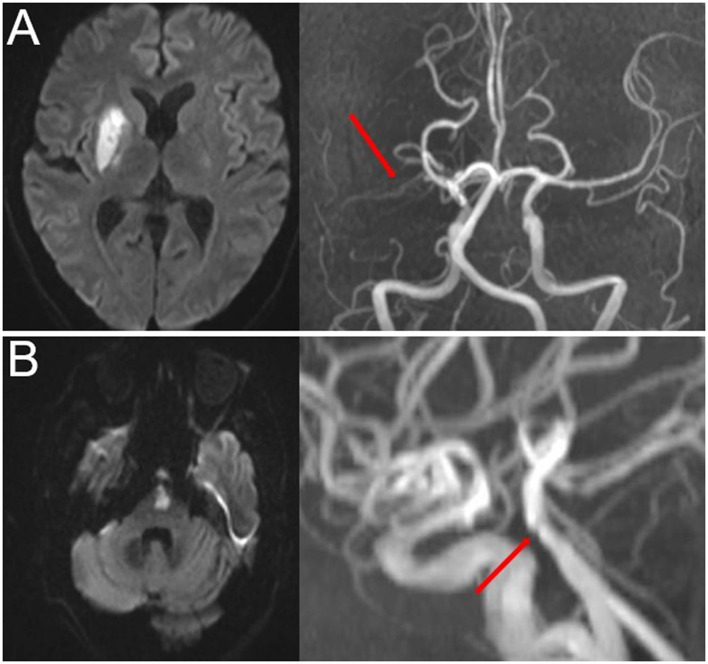
Representative cases of PAD. **(A)** Anterior SSI with PAD (red arrow) and **(B)** posterior SSI with PAD (red arrow). SSI, single subcortical infarction; PAD, parental arterial disease.

Time-of-flight magnetic resonance angiography (TOF-MRA) was used to detect intracranial atherosclerotic stenosis. The most commonly used parameters in TOF-MRA were as follows: flip angle = 20; TR = 30 ms; TE = 10 ms; slice thickness = 1.2 mm; and field of view = 230 mm. The degree of stenosis was evaluated using the Warfarin-Aspirin Symptomatic Intracranial Disease Study Trial method (24). PAD was defined as any degree of stenosis detected in the parent artery corresponding to SSI (15, 21) (Figure 2).

Asymptomatic intracranial stenosis was considered to be a confounding factor affecting functional outcomes. Therefore, we evaluated atherosclerotic conditions of all intracranial large arteries, including the bilateral anterior cerebral, the middle cerebral, the posterior cerebral, the intracranial internal carotid, the intracranial vertebral, and the basilar arteries. Asymptomatic stenosis was defined as a stenosis of ≥ 50% of the intracranial large artery that was not associated with an infarct. The number of asymptomatic intracranial stenoses in each participant was counted. In the case of existence of tandem stenoses in a single artery, asymptomatic intracranial stenosis was counted as 1.

Extracranial stenosis was detected by color Doppler ultrasound (Philips, Netherlands) using a 3.0–9.0 MHz ultrawideband linear array probe. The degree of stenosis was evaluated according to the North American Symptomatic Carotid Endarterectomy Trial method (25).

Two independent radiological or ultrasound experts who were blinded to the baseline characteristics of the participants assessed intracranial and extracranial atherosclerosis, respectively. When these experts disagreed on the assessment, a third superior practitioner made the final diagnosis.

### Follow-up

An independent neurologist who was blinded to the patients' baseline and neuroimaging information contacted participants or their surrogates *via* telephone at 30, 60, and 90 days after the onset of the index stroke. The control of risk factors and the medication compliance of participants were conveyed through a questionnaire survey. In a 90-day follow-up, daily activities of all participants were assessed using the mRS (0–5 points; death was counted as 6 points), and a mRS score of > 2 points was considered to be a 90-day poor functional outcome.

### Statistical analysis

Statistical analyses were performed using SPSS software 25.0 (IBM, Armonk, NY, USA). Categorical variables were compared using the chi-squared test or Fisher's exact test. Normally distributed continuous variables were compared using independent sample *t-*tests, and nonnormally distributed continuous variables were compared using the Mann–Whitney *U*-tests.

Age, sex, and all clinical factors for which a *p*-value of < 0.05 in the univariable analyses were included in the multivariable modified Poisson regression model (26). Multivariable modified Poisson regression analyses were performed to obtain the factors that were independently correlated with the occurrence of ND and a 90-day poor functional outcome in participants and to analyze the correlations between the presence of PAD and worsened NIHSS. A receiver operating characteristic (ROC) curve was used to calculate the area under the curve (AUC) of clinical factors for the prediction of a 90-day poor functional outcome. All tests were two-sided, and a *p-*value of < 0.05 was considered statistically significant.

## Results

**Figure 3:**
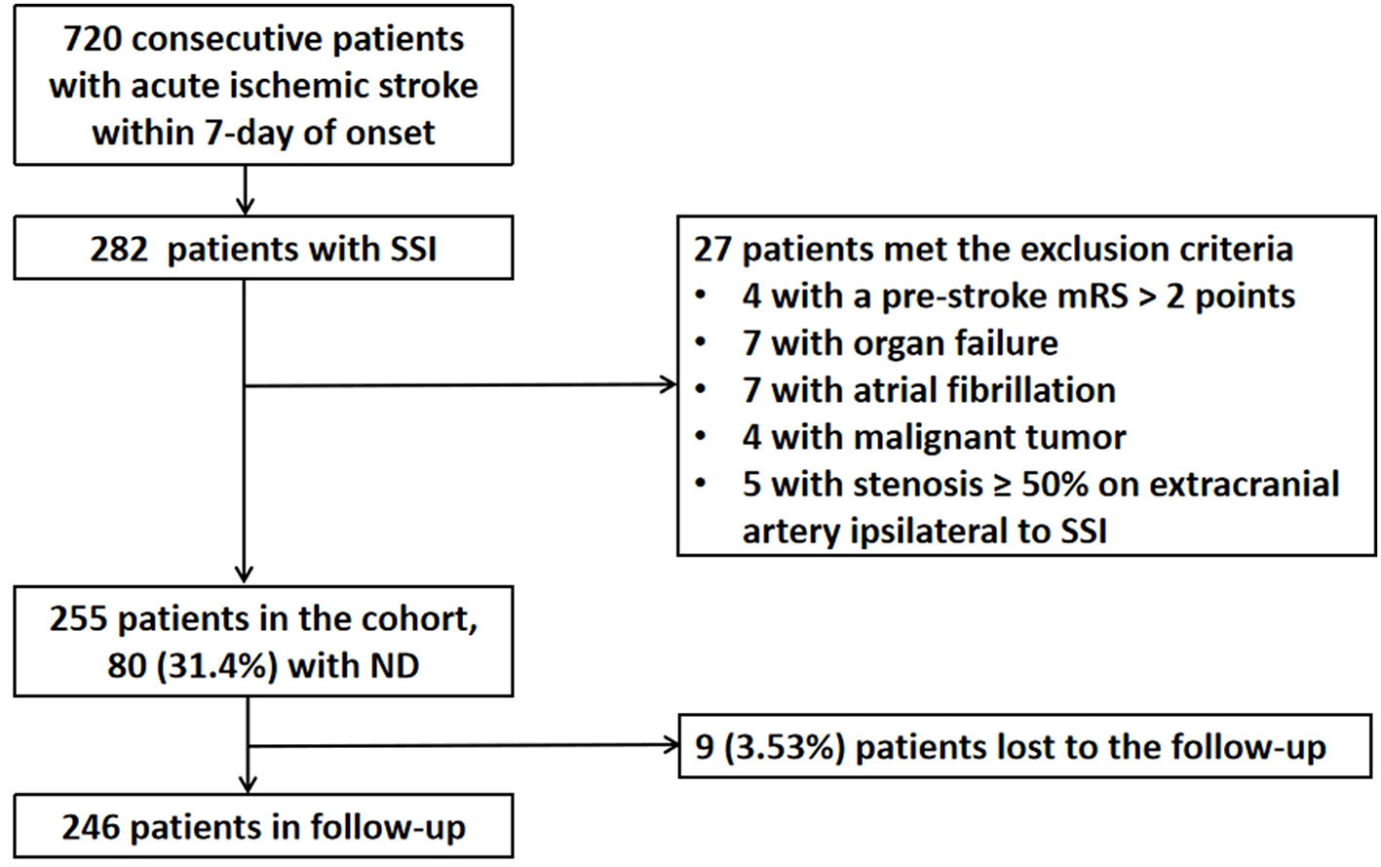
Flowchart of patient enrollment and follow-up. SSI, single subcortical infarction; ND, neurological deterioration; mRS, modified Rankin scale.

A total of 720 patients with ischemic stroke within 7 days of onset were admitted to our stroke unit, and 282 (39.2%) patients were diagnosed with SSI. Of these 282 patients, five patients with a stenosis of ≥ 50% in the extracranial artery ipsilateral to SSI, four patients with a mRS > 2 points before the index stroke, seven patients with organ failure, seven patients with potential cardioembolism, and four patients with malignant tumors were excluded. Of the 255 patients enrolled in the cohort, 80 of them (31.4%) experienced ND during hospitalization. In a 90-day follow-up, nine (3.53%) participants were missing (Figure 3). Of the 246 participants who were retained in the follow-up, 42 (17.1%) had a 90-day poor functional outcome (mRS > 2 points).

### Independent predictors of ND and a 90-day poor functional outcome in patients with SSI

Participants were divided into two groups: those with SSI without ND (SSI-noND) (*n* = 169) and those with SSI-ND (*n* = 77). The SSI-ND group had higher levels of glycosylated hemoglobin and high-sensitivity C-reactive protein (hs-CRP), more asymptomatic intracranial stenoses, a higher proportion of thrombolysis, a longer hospital stay, and a worse 90-day functional outcome than the SSI-noND group (Table 1). In the multivariable modified Poisson regression model, the level of glycosylated hemoglobin [adjusted relative risk (aRR) = 1.13, 95% confidence interval (CI) = 1.04–1.23, *p* = 0.006], asymptomatic intracranial stenosis (aRR= 1.13, 95% CI = 1.003–1.27, *p* = 0.044), and thrombolysis (aRR= 3.75, 95% CI = 2.87–4.91, *p* < 0.001) were the independent predictors of the occurrence of ND (Table 2).

**Table 1:** Clinical characteristics of participants with and without ND.

**Clinical characteristics**	**Total (*n =* 246)**	**SSI-noND (*n =* 169)**	**SSI-ND (*n =* 77)**	***P*-value**
Women, *n* (%)	89 (36.2)	57 (33.3)	32 (41.6)	0.24
Age (years), mean ± SD	64.8 ± 10.7	64.6 ± 10.5	65.2 ± 11.1	0.70
Hypertension, *n* (%)	168 (68.3)	111 (65.7)	57 (74.0)	0.19
Diabetes mellitus, *n* (%)	74 (30.1)	45 (26.6)	29 (37.7)	0.08
Smoking, *n* (%)	107 (43.5)	77 (45.6)	30 (39.0)	0.33
Alcohol consumption, *n* (%)	86 (35.0)	59 (34.9)	27 (35.1)	0.98
Previous stroke, *n* (%)	29 (11.8)	20 (11.8)	9 (11.7)	0.97
BMI, median (IQR)	25.39 (23.26, 27.34)	25.39 (23.37, 27.34)	25.08 (22.73, 27.34)	0.30
SBP (mmHg), median (IQR)	153.5 (142.0, 170.0)	152.0 (142.0, 166.0)	157.0 (142.5, 176.0)	0.22
DBP (mmHg), median (IQR)	85.0 (76.0, 94.0)	85.0 (76.0, 94.0)	85.0 (77.0, 95.5)	0.46
TG (mmol/L), median (IQR)	1.48 (1.08, 2.09)	1.49 (1.02, 2.15)	1.44 (1.12, 1.83)	0.72
TC (mmol/L), mean ± SD	4.62 ± 1.00	4.66 ± 1.03	4.54 ± 0.95	0.37
HDL-C (mmol/L), median (IQR)	1.00 (0.85, 1.25)	0.99 (0.85, 1.22)	1.04 (0.85, 1.31)	0.57
LDL-C (mmol/L), mean ± SD	2.71 ± 0.84	2.74 ± 0.88	2.65 ± 0.75	0.47
Uric acid (mmol/L), median (IQR)	300.95 (251.98, 376.75)	296.20 (252.80, 367.65)	305.20 (240.95, 388.35)	0.87
HbA1c (%), median (IQR)	6.20 (5.80, 7.73)	6.10 (5.80, 7.45)	6.80 (5.85, 8.95)	0.014^*^
Homocysteine (mmol/L), median (IQR)	11.68 (9.29, 14.09)	11.89 (9.37, 14.46)	10.97 (8.94, 13.89)	0.33
WBC (× 10^9^/L), median (IQR)	6.60 (5.50, 8.03)	6.40 (5.40, 8.00)	7.00 (5.70, 8.30)	0.22
Neutrophil count (× 10^9^/L), median (IQR)	4.30 (3.40, 5.50)	4.30 (3.40, 5.20)	4.40 (3.45, 6.05)	0.16
Hs-CRP (mg/L), median (IQR)	1.10 (0.50, 3.33)	1.00 (0.50, 2.60)	1.50 (0.50, 4.30)	0.03^*^
Initial NIHSS (point), median (IQR)	2.0 (1.0, 3.0)	2.0 (1.0, 3.0)	2.0 (1.0, 4.0)	0.74
Asymptomatic stenosis, median (IQR)	1.0 (0.0, 2.0)	1.0 (0.0, 1.0)	1.0 (0.0, 2.0)	0.035^*^
Thrombolysis, *n* (%)	7 (2.8)	0 (0.0)	7 (9.1)	< 0.001^*^
Hospital stay (days), median (IQR)	11.0 (9.0, 16.0)	10.0 (8.0, 12.0)	30.0 (14.5, 49.5)	< 0.001^*^
Regular secondary prevention, *n* (%)	217 (88.2)	149 (88.2)	68 (88.3)	0.97
90-day poor functional outcome, *n* (%)	42 (17.1)	14 (8.3)	28 (36.4)	< 0.001^*^
**Lesion characteristics**				
PAD, *n* (%)	75 (30.5)	51 (30.2)	24 (31.2)	0.88
pSSI, *n* (%)	173 (70.3)	120 (71.0)	53 (68.8)	0.73
Anterior lesion, *n* (%)	172 (69.9)	123 (72.8)	49 (63.6)	0.15

ND, neurological deterioration; SSI-noND, patients with single subcortical infarction without neurological deterioration; SSI-ND, patients with single subcortical infarction with neurological deterioration; BMI, body mass index; SBP, systolic blood pressure; DBP, diastolic blood pressure; TG, triglyceride; TC, total cholesterol; HDL-C, high-density lipoprotein cholesterol; LDL-C, low-density lipoprotein cholesterol; HbA1c, glycosylated hemoglobin; WBC, white blood cell count; hs-CRP, high-sensitivity C-reactive protein; NIHSS, National Institutes of Health Stroke Scale; PAD, parental arterial disease; pSSI, proximal single subcortical infarction.

* p < 0.05 was considered statistically significant.

**Table 2:** Independent predictors of ND in patients with SSI.

**Clinical factors**	**Occurrence of ND**			
	**Crude RR (95% CI)**	***P*** **-value**	**Adjusted RR**^†^ **(95% CI)**	***P-*** **value**
Age	1.001 (0.996–1.007)	0.71	0.998 (0.98–1.02)	0.83
Women	1.08 (0.95–1.22)	0.24	1.20 (0.84–1.72)	0.32
HbA1c (%)	1.04 (1.01–1.08)	0.026^*^	1.13 (1.04–1.23)	0.006^*^
Asymptomatic stenosis	1.05 (1.001–1.092)	0.049^*^	1.13 (1.003–1.27)	0.044^*^
Thrombolysis	2.03 (1.91–2.15)	< 0.001^*^	3.75 (2.87–4.91)	< 0.001^*^

† Adjusted for HbA1c, hs-CRP, asymptomatic stenosis, and thrombolysis. ND, neurological deterioration; SSI, single subcortical infarction; HbA1c, glycosylated hemoglobin; hs-CRP, high-sensitivity C-reactive protein.

* p < 0.05 was considered statistically significant.

Patients with SSI were classified as good and poor outcome groups based on their functional outcomes. After adjusting for variables that showed significant differences in univariable analyses (including sex, a history of diabetes mellitus, smoking, alcohol consumption, systolic blood pressure on admission, glycosylated hemoglobin, hs-CRP, initial NIHSS, ND, hospital stay, PAD, and pSSI; Supplementary Table S1), female sex (aRR = 1.13, 95% CI = 1.03–1.24, *p* = 0.012), initial NIHSS score (aRR = 1.08, 95% CI = 1.07–1.10, *p* < 0.001), ND (aRR = 1.37, 95% CI = 1.22–1.55, *p* < 0.001), and the presence of PAD (aRR= 1.16, 95% CI = 1.07–1.26, *p* = 0.038) were independently associated with a 90-day poor functional outcome in patients with SSI (Table 3).

**Table 3:** Independent predictors of a 90-day poor functional outcome in patients with SSI.

**Clinical factors**	**90-day poor functional outcome**			
	**Crude RR (95% CI)**	***P*** **-value**	**Adjusted RR**^†^ **(95% CI)**	***P-*** **value**
Age	1.004 (0.999–1.009)	0.11	1.00 (0.998–1.004)	0.97
Women	1.23 (1.11–1.37)	< 0.001^*^	1.13 (1.03–1.24)	0.012^*^
Initial NIHSS	1.09 (1.07–1.11)	< 0.001^*^	1.08 (1.07–1.10)	< 0.001^*^
ND	1.32 (1.18–1.49)	< 0.001^*^	1.37 (1.22–1.55)	< 0.001^*^
PAD	1.26 (1.13–1.42)	< 0.001^*^	1.16 (1.07–1.26)	< 0.001^*^

† Adjusted for a history of diabetes mellitus, smoking, alcohol consumption, SBP, HbA1c, hs-CRP, initial NIHSS, ND, hospital stay, PAD, and pSSI. SSI, single subcortical infarction; SBP, systolic blood pressure; HbA1c, glycosylated hemoglobin; hs-CRP, high-sensitivity C-reactive protein; NIHSS, National Institutes of Health Stroke Scale; ND, neurological deterioration; PAD, parental arterial disease; pSSI, proximal single subcortical infarction.

* p < 0.05 was considered statistically significant.

### Independent predictors of poor outcome in patients with SSI-ND

The SSI-ND subgroup was classified into two groups: those with a 90-day good functional outcome and those with a 90-day poor functional outcome. Compared to the good outcome group, in the poor outcome group, the proportion of women, the proportion of individuals with PAD, and the levels of glycosylated hemoglobin and hs-CRP were higher, the NIHSS score was highest, asymptomatic intracranial stenosis was present, the age of the participants was higher (i.e., older adults), and the proportion of patients who consumed alcohol was lower (all *p* < 0.05) (Supplementary Table S2).

The aforementioned factors that showed significant differences in univariable analyses were included in modified Poisson regression analyses. The level of glycosylated hemoglobin [aRR = 1.17, 95% CI = 1.01–1.35, *p* = 0.035], the most severe NIHSS score (aRR= 1.15, 95% CI = 1.06–1.25, *p* = 0.001), and the presence of PAD (aRR= 2.15, 95% CI = 1.20–3.86, *p* = 0.01) could independently predict a 90-day poor functional outcome in patients with SSI-ND (Table 4).

**Table 4:** Independent predictors of a 90-day poor functional outcome in patients with SSI-ND.

**Clinical factors**	**90-day poor functional outcome**			
	**Adjusted RR**^†^ **(95% CI)**	**P value**	**Adjusted RR**^†^ **(95% CI)**	***P*** **-value**
Women	2.97 (1.55–5.69)	0.001^*^	2.40 (0.75–7.70)	0.14
Age	1.03 (1.004–1.06)	0.025^*^	1.002 (0.97–1.03)	0.89
Alcohol consumption	0.40 (0.17–0.94)	0.035^*^	1.14 (0.44–2.93)	0.79
HbA1c	1.23 (1.09–1.38)	0.001^*^	1.17 (1.01–1.35)	0.035^*^
Hs-CRP	1.01 (0.99–1.02)	0.31	1.01 (0.996–1.02)	0.18
More severe NIHSS	1.17 (1.10–1.25)	< 0.001^*^	1.15 (1.06–1.25)	0.001^*^
Asymptomatic stenosis	1.19 (1.01–1.41)	0.037^*^	1.02 (0.89–1.16)	0.80
PAD	3.98 (2.17–7.27)	< 0.001^*^	2.15 (1.20–3.86)	0.01^*^

† Adjusting for alcohol consumption, HbA1c, hs-CRP, more severe NIHSS, asymptomatic stenosis, and the presence of PAD. SSI-ND indicates patients with single subcortical infarction with neurological deterioration; HbA1c, glycosylated hemoglobin; hs-CRP, high-sensitivity C-reactive protein; NIHSS, National Institutes of Health Stroke Scale; PAD, parental arterial disease.

* p < 0.05 was considered statistically significant.

### Comparison of NIHSS profiles of patients with SSI-ND with and without PAD

**Figure 4:**
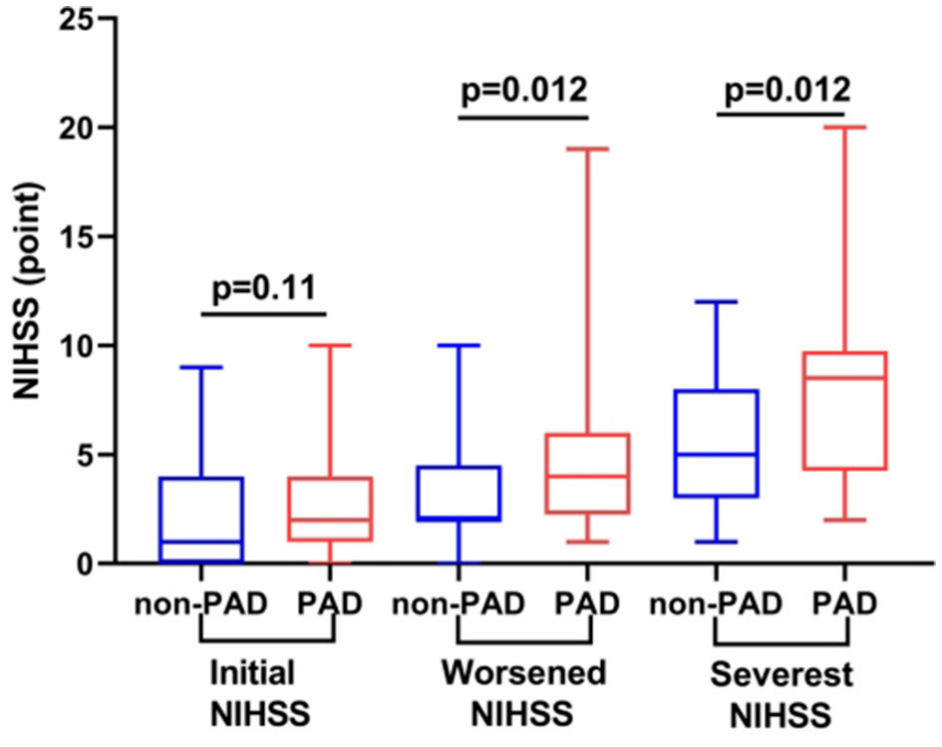
Comparison of the NIHSS profiles of participants with and without PAD. Compared with participants without PAD, those with PAD had comparable initial NIHSS scores but higher/worsened and higher/more severe NIHSS scores (both *p* = 0.012). NIHSS, National Institutes of Health Stroke Scale; PAD, parental arterial disease.

Of 77 patients with SSI-ND during a follow-up, 24 of them (31.2%) had PAD. Based on the presence of PAD, all participants were divided into the PAD and non-PAD groups. Initial NIHSS scores of these two groups were comparable [2.0 (1.0, 4.0) vs. 1.0 (0.0, 4.0) points, *p* = 0.11]. However, the PAD group had a higher/worsened score on the NIHSS [4.0 (2.3, 6.0) vs. 2.0 (2.0, 4.5) points, *p* = 0.012] and a higher/most severe score on the NIHSS [8.5 (4.3, 9.8) vs. 5.0 (3.0, 8.0) points, *p* = 0.012] than the non-PAD group (Figure 4).

### Correlation between the presence of PAD and a higher/worsened NIHSS in patients with SSI-ND

Based on the median values, the NIHSS of the 77 patients with SSI-ND was worsened by [2.0 (2.0, 5.0)] points. Participants were dichotomized into two groups: those with a lower/ worsened NIHSS (≤ 2 points) and those with a higher/worsened NIHSS (> 2 points). In univariable analyses, the higher/worsened NIHSS group had a larger percentage of PAD than the lower/worsened NIHSS group (47.4 vs. 15.4%, *p* = 0.002, Supplementary Table S3).

In multivariable-modified Poisson regression, after adjusting for age and sex, the presence of PAD (aRR = 1.87, 95% CI = 1.19–2.95, *p* = 0.007) was independently correlated with a higher/worsened NIHSS (data not shown).

### Clinical factors were independently correlated with a 90-day poor functional outcome in patients with SSI-ND with PAD

Approximately 24 patients with SSI-ND with PAD were divided into two groups: patients with a 90-day good functional outcome (*n* = 6) and patients with a 90-day poor functional outcome (*n* = 18). In univariable analyses, compared with the good functional outcome group, the the poor functional outcome group had significantly higher NIHSS profiles, including an initial NIHSS [3.0 (2.0, 5.5) vs. 1.0 (0.0, 1.5) points, *p* = 0.007], a worsened NIHSS [4.5 (3.0, 7.0) vs. 2.5 (1.8, 4.3) points, *p* = 0.047], and the most severe NIHSS (9.3 ± 3.8 vs. 3.8 ± 2.2 points, *p* = 0.003) scores (Supplementary Table S4).

When NIHSS profiles, age, and sex were simultaneously included in the modified Poisson regression model, only the most severe NIHSS (aRR = 1.09, 95% CI = 1.02–1.17, *p* = 0.01) was independently correlated with a 90-day poor functional outcome in patients with SSI-ND with PAD (Table 5).

**Table 5:** Independent predictors of a 90-day poor functional outcome in patients with SSI-ND with PAD.

**NIHSS profile**	**90-day poor functional outcome**			
	**Model 1** ^†^		**Model 2** ^‡^	
	**Adjusted RR (95% CI)**	***P*** **-value**	**Adjusted RR (95% CI)**	***P-*** **value**
Initial NIHSS	1.11 (1.02–1.20)	0.013^*^	1.05 (0.97–1.12)	0.22
Worsened NIHSS	1.06 (1.005–1.12)	0.033^*^	–	–
More severe NIHSS	1.10 (1.03–1.18)	0.008^*^	1.09 (1.02–1.17)	0.015^*^

† Adjusted for age and sex.

‡ NIHSS profiles, age, and sex were simultaneously included in the multivariable-modified Poisson regression model. SSI-ND indicates patients with single subcortical infarction with neurological deterioration; PAD, parental arterial disease; NIHSS, National Institutes of Health Stroke Scale.

* p < 0.05 was considered statistically significant.

### Predictive AUC of NIHSS profiles using an ROC analysis in patients with SSI-ND with PAD

**Figure 5:**
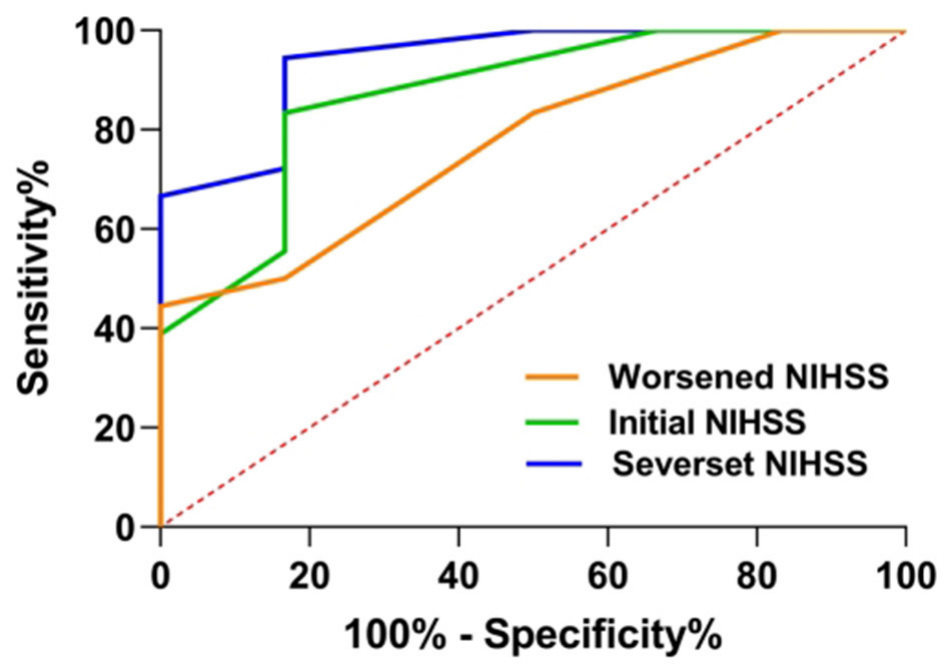
A receiver operating characteristic (ROC) curve of the NIHSS profiles was used to predict a 90-day poor functional outcome in patients with SSI-ND with PAD. NIHSS, National Institutes of Health Stroke Scale; SSI-ND, patients with single subcortical infarction with neurological deterioration; PAD, parental arterial disease.

In predicting a 90-day poor functional outcome in patients with SSI-ND with PAD, after adjusting for age and sex, the most severe NIHSS score had the largest AUC (0.94, 95% CI = 0.83–1.00, *p* = 0.002), followed by an initial NIHSS (AUC = 0.87, 95% CI = 0.70–1.00, *p* = 0.008) and a worsened NIHSS score (AUC = 0.77, 95% CI = 0.57–0.98, *p* = 0.049) (Figure 5). The predictive cutoff for the most severe NIHSS for a 90-day poor functional outcome in patients with SSI-ND with PAD was 4.5 points, with a sensitivity of 94.0% and a specificity of 83.3%.

## Discussion

In this study, we found that ND is an independent predictor of poor outcomes in patients with SSI. Clinical factors that predicted a poor outcome in the group of all patients with SSI were different from those that predicted a poor outcome in the SSI-ND subgroup. For patients with SSI-ND, the presence of PAD was independently correlated with a poor outcome and a higher/worsened NIHSS. For the SSI-ND subgroup with PAD, the most severe NIHSS score was the only independent predictor of a 90-day poor functional outcome, with a predictive cutoff of 4.5 points; a sensitivity of 94.0%, and a specificity of 83.3%.

Because patients with SSI are more likely to develop ND than patients with other stroke subtypes (6–9) and the existence of a clear effect of ND on functional recovery after SSI is unknown, we specifically explored the effect of ND on functional outcomes in patients with SSI. ND was found to be an independent predictor of poor functional outcomes in patients with SSI (Table 3). It is suggested that patients with SSI-ND tend to have a worse outcome than those with SSI-noND, which is consistent with our conjecture. Other predictors of a poor outcome in patients with SSI are female sex, initial NIHSS, and the presence of PAD, which are consistent with the findings reported in previous studies (16, 27).

Given that the SSI-ND subgroup had a higher proportion of patients with a poor outcome than the SSI group (36.4% vs. 17.1%), clinical factors that predicted a poor outcome in the SSI-ND subgroup might differ from those that predicted a poor outcome in the SSI group. Subsequently, we focused on poor outcome determinants in the SSI-ND subgroup and found that the level of glycosylated hemoglobin, a more severe NIHSS, and the presence of PAD independently predict poor outcomes in patients with SSI-ND. These determinants are completely different from poor outcome predictors in the entire SSI population. It was suggested that there are differences between the SSI-ND subgroup and the SSI group in terms of parameters that allow early identification of individuals who are at a high risk of achieving poor outcomes and the type of management that will improve functional outcomes.

In the SSI-ND subgroup, the presence of PAD and a severe NIHSS score were the two independent predictors of a poor functional outcome. We investigated the relationships between the presence of PAD and participants' NIHSS profiles, including their initial, worsened, and more severe NIHSS scores. We divided patients with SSI-ND into groups with and without PAD and found that the initial NIHSS scores of these two groups were comparable. However, compared with the group without PAD, both worsened and more severe NIHSS scores were significantly higher in the PAD group. In multivariable analyses, the presence of PAD was independently associated with a higher/worsened NIHSS score [> 2 points (median)]. These results indicate that the presence of PAD can aggravate the degree of ND, thus worsening stroke severity and ultimately affecting functional outcomes in patients with SSI-ND. Therefore, the presence of PAD is probably an essential factor that, at least in part, determines functional outcomes in patients with SSI-ND.

The effect of PAD on the degree to which ND occurs is probably due to the different mechanisms through which infarcts form in patients with and without PAD. In patients with SSI with PAD, infarct formation derives from the orifice of the perforating artery blocked by an atheroma in the parent artery (1), whereas in patients without PAD, infarct formation is attributed to lipohyalinosis or fibrinoid degeneration of the perforating artery (2). In cases where there is progression or rupture of the atheroma in the parent artery, patients with SSI with PAD are likely to experience ND (15). Pathological progress associated with the progression of an atheroma could completely block the orifice of the perforating artery, causing ischemia throughout the supply territory. For patients with SSI without PAD, ND is most likely caused by the progression of perforating artery disease, resulting in mild ischemia in the supply territory. Therefore, ND in patients with SSI with PAD is likely to be more severe than that in patients without PAD.

Although the majority (18/24, 75%) of patients with SSI-ND with PAD had a poor functional outcome, there were still a few patients with a good outcome. We compared the clinical characteristics of patients with different outcomes and found that initial, worsened, and more severe NIHSS scores differed significantly between the two groups. In multivariable analyses, only a more severe NIHSS score was independently associated with a 90-day poor functional outcome in patients with SSI-ND with PAD. Thereafter, we performed ROC analyses and concluded that, for patients with SSI-ND with PAD, a more severe NIHSS score > 4 points is associated with an extremely high risk of a poor functional outcome.

In this study, the level of glycosylated hemoglobin was another independent factor determining a 90-day functional outcome in patients with SSI-ND. The mechanism underlying this effect is that high plasma glucose contributes to increased blood pressure and the upregulation of inflammatory cytokines (28, 29), thus hindering functional recovery. For patients with SSI-ND, plasma glucose needs to be intensively managed.

According to previous research, SSI can be classified into pSSI and dSSI based on the positional relationship between SSI and the parent artery (21). A number of studies have demonstrated that patients with pSSI have larger infarct sizes, more severe neurological deficits, and a worse functional outcome than patients with dSSI (4, 30). However, in this study, the positional relationship between SSI and the parent artery was not associated with functional outcomes in patients with SSI. This discrepancy may be due to differences in study design, study population, and auxiliary examination methods between this study and previous studies.

In univariable analysis, the proportion of patients in the subgroup with SSI-ND who consumed alcohol was lower in the poor outcome group than in the good outcome group. A previous study reported that the prestroke function of alcohol consumers was better than that of non-alcohol consumers (31), which is consistent with the present study to some extent. This interesting phenomenon may involve the protection of neurons, stabilization of the blood–brain barrier, and attenuation of reperfusion injury after ischemic stroke with ethanol (32, 33). In addition, participants with poor functional outcomes had higher levels of hs-CRP than those with good outcomes, which was consistent with a previous study (34).

We also explored the factors associated with the occurrence of ND in patients with SSI. The level of glycosylated hemoglobin, asymptomatic intracranial stenosis, and thrombolysis were all independently correlated with ND in patients with SSI. This result is consistent with a previous study (16, 35, 36). In a univariable analysis, we found that patients with SSI-ND had higher hs-CRP levels than those without ND. However, in multivariable regression, hs-CRP was not an independent predictor of the occurrence of ND in patients with SSI. Duan et al. have recently reported that hs-CRP can predict ND in patients with stroke with atrial fibrillation but not in patients with stroke without atrial fibrillation (37). These results suggest that hs-CRP may not be applicable to predict the occurrence of ND in patients with stroke.

This study has several limitations. First, it is a single-center study with a relatively small sample size, especially with respect to the SSI-ND subgroup with PAD. Another study with a larger sample size should be conducted to validate the results of this study. Second, this study enrolled only patients with SSI who experienced ND after admission. It was likely that some patients experienced ND before admission, but these patients were excluded because their ND could not be verified. Therefore, in this study, there might be a selection bias in the recruitment of participants. Third, TOF-MRA was used to assess intracranial stenosis. This traditional MRA is prone to artifacts caused by blood flow abnormalities, and the assessment of stenosis may be hampered by blood flow velocity (38). Due to the noninvasiveness and accessibility of TOF-MRA, it is still widely used in the evaluation of intracranial atherosclerosis.

## Conclusion

Neurological deterioration is an independent predictor of a poor functional outcome in patients with SSI. Poor outcome determinants in the SSI-ND subgroup differ from those in all patients with SSI. In the SSI-ND subgroup, the presence of PAD may aggravate the degree of ND, thus worsening stroke severity. Therefore, PAD is most likely an essential factor in determining functional outcomes. The most severe NIHSS score is the only determinant of functional outcomes in patients with SSI-ND with PAD, and those with the most severe NIHSS score of > 4 points are at an extremely high risk of a poor functional outcome.

## Data availability statement

The raw data supporting the conclusions of this article will be made available by the authors, without undue reservation.

## Ethics statement

The studies involving human participants were reviewed and approved by the Medical Research Ethics Committee of Affiliated Hospital of Jiangsu University. The patients/participants provided their written informed consent to participate in this study.

## Author contributions

YY and YH: design and writing. WH and JX: visualization. ZC, TZ, and YS: methods and resources. YY: revising. MY: design, revising, reviewing, supervision, and editing of final version of the manuscript. All authors contributed to the article and approved the submitted version.
