# Supplementary material for: Clinical factors associated with functional outcomes in patients with single subcortical infarction with neurological deterioration
Source: Front Neurol. 2023 Mar 23;14:1129503. doi: 10.3389/fneur.2023.1129503 (PMC10077891; doi:10.3389/fneur.2023.1129503)
Supplement: Supplementary file 1 [file Table_1.DOCX]

Supplementary Table 1. Comparisons of clinical factors based on different functional outcomes

| Clinical characteristics | Good outcome (n=204) | Poor outcome (n=42) | P-value |
| --- | --- | --- | --- |
| Female, n (%) | 62 (30.4) | 27 (64.3) | <0.001^*^ |
| Age (year), mean ± SD | 64.2±10.3 | 67.4±12.2 | 0.077 |
| Hypertension, n (%) | 138 (67.6) | 30 (71.4) | 0.63 |
| Diabetes mellitus, n (%) | 56 (27.5) | 18 (42.9) | 0.047^*^ |
| Smoking, n (%) | 95 (46.6) | 12 (28.6) | 0.032^*^ |
| Alcohol consumption, n (%) | 78 (38.2) | 8 (19.0) | 0.018^*^ |
| Previous stroke, n (%) | 24 (11.8) | 5 (11.9) | 1.00 |
| BMI, median (IQR) | 25.39 (23.43, 27.34) | 25.25 (22.13, 27.34) | 0.30 |
| SBP (mmHg), median (IQR) | 152.5 (142.0, 166.0) | 163.0 (146.8, 176.0) | 0.047^*^ |
| DBP (mmHg), median (IQR) | 85.0 (76.3, 94.0) | 85.0 (74.0, 94.0) | 0.46 |
| TG (mmol/L), median (IQR) | 1.47 (1.07, 2.15) | 1.48 (1.08, 1.81) | 0.58 |
| TC (mmol/L), mean ± SD | 4.61±0.97 | 4.69±1.17 | 0.62 |
| HDL-C (mmol/L), median (IQR) | 1.00 (0.84, 1.24) | 1.04 (0.86, 1.33) | 0.54 |
| LDL-C (mmol/L), mean ± SD | 2.70±0.82 | 2.77±0.93 | 0.59 |
| Uric acid (mmol/L), median (IQR) | 305.10 (257.03, 383.25) | 287.85 (233.30, 351.13) | 0.17 |
| HbA1c (%), median (IQR) | 6.10 (5.80, 7.50) | 6.70 (5.88, 10.18) | 0.019^*^ |
| Homocysteine (mmol/L), median (IQR) | 11.81 (9.34, 14.43) | 11.05 (8.90, 13.64) | 0.31 |
| WBC (×10^9^/L), median (IQR) | 6.50 (5.50, 7.90) | 7.10 (5.85, 8.70) | 0.11 |
| Neutrophil count (×10^9^/L), median (IQR) | 4.30 (3.40, 5.40) | 4.50 (3.40, 6.13) | 0.23 |
| Hs-CRP (mg/L), median (IQR) | 1.00 (0.50, 2.60) | 2.30 (0.78, 4.25) | 0.007^*^ |
| Initial NIHSS (point), median (IQR) | 2.0 (1.0, 2.0) | 5.0 (2.0, 7.0) | <0.001^*^ |
| ND, n (%) | 49 (24.0) | 28 (66.7) | <0.001^*^ |
| Asymptomatic stenosis, median (IQR) | 1.0 (0.0, 2.0) | 1.0 (0.0, 2.3) | 0.077 |
| Thromblysis, n (%) | 5 (2.5) | 2 (4.8) | 0.76 |
| Hospital stay (day), median (IQR) | 10.0 (8.0, 13.0) | 17.0 (13.0, 29.5) | <0.001^*^ |
| Regular secondary prevention, n (%) | 181 (88.7) | 36 (85.7) | 0.77 |
| Lesional characteristics |  |  |  |
| PAD, n (%) | 50 (24.5) | 25 (59.5) | <0.001^*^ |
| pSSI, n (%) | 137 (67.2) | 36 (85.7) | 0.017^*^ |
| Anterior lesion, n (%) | 142 (69.6) | 30 (71.4) | 0.82 |

Abbreviations: SSI-ND indicates patients with single subcortical infarction with neurologic deterioration, BMI, body mass index; SBP, systolic blood pressure; DBP, diastolic blood pressure; TG, triglyceride; TC, total cholesterol; HDL-C, high-density lipoprotein-cholesterol; LDL-C, low-density lipoprotein cholesterol; HbA1c, glycosylated hemoglobin; WBC, white blood cell count; Hs-CRP, high-sensitivity C-reactive protein; NIHSS, National Institute of Health Stroke Scale; ND indicates neurological deterioration; PAD, parent artery disease; pSSI, proximal single subcortical infarction.

* *p* < 0.05 was considered statistically significant.

**Supplementary Table 2. Comparisons of clinical factors based on different functional outcomes in subgroup of SSI-ND**

| Clinical factors | SSI-ND | | |
| --- | --- | --- | --- |
|  | Good outcome (n=49) | Poor outcome (n=28) | P-value |
| Female, n (%) | 13 (26.5) | 19 (67.9) | <0.001^*^ |
| Age (year), mean ± SD | 63.0±10.0 | 69.0±12.1 | 0.023^*^ |
| Hypertension, n (%) | 36 (73.5) | 21 (75.0) | 0.88 |
| Diabetes mellitus, n (%) | 15 (30.6) | 14 (50.0) | 0.091 |
| Smoking, n (%) | 22 (44.9) | 8 (28.6) | 0.16 |
| Alcohol consumption, n (%) | 22 (44.9) | 5 (17.9) | 0.017^*^ |
| Previous stroke, n (%) | 6 (12.2) | 3 (10.7) | 1.00 |
| BMI, median (IQR) | 25.08 (23.43, 27.34) | 24.94 (22.29, 27.17) | 0.50 |
| SBP (mmHg), mean ± SD | 157.1±21.9 | 162.5±23.0 | 0.31 |
| DBP (mmHg), mean ± SD | 88.2±14.2 | 85.0±12.5 | 0.33 |
| TG (mmol/L), median (IQR) | 1.28 (1.06, 1.80) | 1.53 (1.18, 1.86) | 0.26 |
| TC (mmol/L), mean ± SD | 4.53±0.87 | 4.54±1.09 | 0.97 |
| HDL-C (mmol/L), median (IQR) | 1.04 (0.87, 1.31) | 0.97 (0.82, 1.32) | 0.49 |
| LDL-C (mmol/L), mean ± SD | 2.64±0.65 | 2.67±0.91 | 0.92 |
| Uric acid (mmol/L), mean ± SD | 321.87±111.53 | 291.38±92.69 | 0.23 |
| HbA1c (%), median (IQR) | 6.40 (5.80, 7.80) | 7.31 (6.23, 10.68) | 0.014^*^ |
| Homocysteine (mmol/L),  median (IQR) | 10.84  (9.11, 13.81) | 11.71  (8.70, 13.90) | 0.97 |
| WBC (×10^9^/L), median (IQR) | 6.9 (5.6, 8.0) | 7.3 (5.9, 9.1) | 0.28 |
| Neutrophil count (×10^9^/L), median (IQR) | 4.3 (3.5, 5.8) | 4.5 (3.5, 6.4) | 0.44 |
| Hs-CRP (mg/L), median (IQR) | 1.10 (0.50, 3.00) | 3.20 (1.08, 6.38) | 0.015^*^ |
| Severest (or intial) NIHSS (point), median (IQR) | 4.0 (2.5, 6.0) | 9.0 (6.0, 11.5) | <0.001^*^ |
| Asymptomatic stenosis, median (IQR) | 1.0 (0.0, 2.0) | 1.0 (1.0, 3.0) | 0.035^*^ |
| END, n (%) | 44 (89.8) | 21 (75.0) | 0.16 |
| Thromblysis, n (%) | 5 (10.2) | 2 (7.1) | 1.00 |
| Hospital stay (day), median (IQR) | 38.0 (12.0, 53.5) | 24.0 (16.3, 34.8) | 0.51 |
| Regular secondary prevention, n (%) | 45 (91.8) | 23 (82.1) | 0.37 |
| Lesional characteristics |  |  |  |
| PAD, n (%) | 6 (12.2) | 18 (64.3) | <0.001^*^ |
| pSSI, n (%) | 31 (63.3) | 22 (78.6) | 0.16 |
| Anterior lesion, n (%) | 28 (57.1) | 21 (75.0) | 0.12 |

Abbreviations: mRS indicates modified Rankin Scale; BMI, body mass index; SBP, systolic blood pressure; DBP, diastolic blood pressure; TG, triglyceride; TC, total cholesterol; HDL-C, high-density lipoprotein-cholesterol; LDL-C, low-density lipoprotein cholesterol; HbA1c, glycosylated hemoglobin; WBC, white blood cell count; NLR, neutrophil to lymphocyte ratio; Hs-CRP, high-sensitivity C-reactive protein; NIHSS, National Institute of Health Stroke Scale; END, early neurological deterioration; PAD, parent artery disease; pSSI, proximal single subcortical infarction.

* *p* < 0.05 was considered statistically significant.

**Supplementary Table 3. Comparisons between SSI-ND patients with lower and higher worsened NIHSS**

| Clinical characteristics | Lower worsened NIHSS (n=39) | Higher worsened NIHSS (n=38) | P-value |
| --- | --- | --- | --- |
| Female, n (%) | 27 (69.2) | 18 (47.4) | 0.052 |
| Age (year), mean ± SD | 65.2±10.1 | 65.2±12.2 | 0.99 |
| Hypertension, n (%) | 31 (79.5) | 26 (68.4) | 0.27 |
| Diabetes mellitus, n (%) | 12 (30.8) | 17 (44.7) | 0.21 |
| Smoking, n (%) | 16 (41.0) | 14 (36.8) | 0.71 |
| Alcohol consumption, n (%) | 14 (35.9) | 13 (34.2) | 0.88 |
| Previous stroke, n (%) | 4 (10.3) | 0 (0.0) | 0.12 |
| BMI, median (IQR) | 25.28 (23.43, 27.68) | 24.79 (22.42, 26.13) | 0.39 |
| SBP (mmHg), mean ± SD | 159.1±23.1 | 159.0±21.7 | 0.99 |
| DBP (mmHg), mean ± SD | 87.5±14.1 | 86.5±13.3 | 0.76 |
| TG (mmol/L), median (IQR) | 1.24 (0.96, 1.80) | 1.50 (1.20, 1.89) | 0.14 |
| TC (mmol/L), mean ± SD | 4.36±0.83 | 4.72±1.04 | 0.10 |
| HDL-C (mmol/L), median (IQR) | 1.02 (0.81, 1.20) | 1.06 (0.87, 1.39) | 0.24 |
| LDL-C (mmol/L), mean ± SD | 2.55±0.58 | 2.76±0.89 | 0.21 |
| Uric acid (mmol/L), mean ± SD | 320.05±118.17 | 301.27±91.27 | 0.44 |
| HbA1c (%), median (IQR) | 6.10 (5.70, 8.80) | 7.31 (6.28, 9.10) | 0.064 |
| Homocysteine (mmol/L), median (IQR) | 12.17 (8.91, 13.90) | 10.72 (8.94, 13.25) | 0.50 |
| WBC (×10^9^/L), median (IQR) | 6.40 (5.50, 8.20) | 7.15 (5.98, 8.70) | 0.27 |
| Neutrophil count (×10^9^/L), median (IQR) | 4.20 (3.40, 6.20) | 4.93 (3.48, 5.95) | 0.45 |
| NLR, median (IQR) | 2.58 (2.06, 4.20) | 2.88 (1.97, 3.97) | 0.82 |
| Hs-CRP (mg/L), median (IQR) | 1.20 (0.50, 3.90) | 1.85 (0.68, 4.68) | 0.24 |
| Asymptomatic stenosis, median (IQR) | 1.0 (0.0, 2.0) | 1.0 (0.0, 2.0) | 0.48 |
| END, n (%) | 32 (82.1) | 33 (86.8) | 0.56 |
| Thromblysis, n (%) | 3 (7.7) | 4 (10.5) | 0.71 |
| pSSI, n (%) | 25 (64.1) | 28 (73.7) | 0.36 |
| Presence of PAD, n (%) | 6 (15.4) | 18 (47.4) | 0.002^*^ |

Abbreviations: SSI-ND indicates patients with single subcortical infarction with neurologic deterioration; NIHSS, National Institute of Health Stroke Scale; BMI, body mass index; SBP, systolic blood pressure; DBP, diastolic blood pressure; TG, triglyceride; TC, total cholesterol; HDL-C, high-density lipoprotein-cholesterol; LDL-C, low-density lipoprotein cholesterol; HbA1c, glycosylated hemoglobin; WBC, white blood cell count; NLR, neutrophil to lymphocyte ratio; Hs-CRP, high-sensitivity C-reactive protein; END, early neurological deterioration; pSSI, proximal single subcortical infarction; PAD, parent artery disease.

* *p* < 0.05 was considered statistically significant.

**Supplementary Table 4. Comparisons of SSI-ND patients with PAD based on different functional outcomes**

| Clinical characteristics | Patients with SSI-ND with PAD | | P-value |
| --- | --- | --- | --- |
|  | Good outcome  (mRS ≤ 2) (n=6) | Poor outcome  (mRS > 2) (n=18) |  |
| Female, n (%) | 3 (50.0) | 13 (72.2) | 0.36 |
| Age (year), median (IQR) | 69.0 (61.5, 75.3) | 74.0 (63.0, 77.0) | 0.57 |
| Hypertension, n (%) | 3 (50.0) | 15 (83.3) | 0.14 |
| Diabetes mellitus, n (%) | 1 (16.7) | 8 (44.4) | 0.35 |
| Smoking, n (%) | 1 (16.7) | 5 (27.8) | 1.00 |
| Alcohol consumption, n (%) | 1 (16.7) | 2 (11.1) | 1.00 |
| Previous stroke, n (%) | 0 (0.0) | 2 (11.1) | 1.00 |
| BMI, mean ± SD | 25.62±5.48 | 24.99±4.12 | 0.77 |
| SBP (mmHg), mean ± SD | 157.3±22.5 | 162.2±24.6 | 0.67 |
| DBP (mmHg), mean ± SD | 85.0±5.9 | 87.4±10.9 | 0.61 |
| TG (mmol/L), mean ± SD | 1.56±0.70 | 1.69±0.75 | 0.71 |
| TC (mmol/L), mean ± SD | 4.62±0.97 | 4.52±1.09 | 0.85 |
| HDL-C (mmol/L), mean ± SD | 1.28±0.39 | 1.11±0.31 | 0.30 |
| LDL-C (mmol/L), mean ± SD | 2.64±0.75 | 2.62±0.86 | 0.95 |
| Uric acid (mmol/L), mean ± SD | 329.22±76.52 | 296.41±102.45 | 0.48 |
| HbA1c (%), median (IQR) | 6.80 (5.63, 8.15) | 6.80 (6.15, 9.35) | 0.53 |
| Homocysteine (mmol/L), median (IQR) | 10.71 (9.42, 14.14) | 12.23 (8.85, 15.09) | 0.69 |
| WBC (×10^9^/L), mean ± SD | 6.6±1.1 | 7.5±2.4 | 0.36 |
| Neutrophil count (×10^9^/L), median (IQR) | 4.5 (3.6, 5.4) | 4.6 (3.3, 6.0) | 0.58 |
| NLR, median (IQR) | 2.89 (2.10, 3.86) | 2.75 (1.91, 7.06) | 0.95 |
| Hs-CRP (mg/L), median (IQR) | 1.70 (0.43, 4.00) | 3.65 (1.10, 10.95) | 0.22 |
| Asymptomatic stenosis, median (IQR) | 1.0 (0.0, 1.3) | 1.0 (1.0, 3.0) | 0.19 |
| END, n (%) | 5 (83.3) | 12 (66.7) | 0.63 |
| Thromblysis, n (%) | 0 (0.0) | 2 (11.1) | 1.00 |
| Hospital stay (day), mean ± SD | 23.5±8.1 | 21.9±6.5 | 0.63 |
| Regular secondary prevention, n (%) | 5 (83.3) | 14 (77.8) | 1.00 |
| Lesional characteristics |  |  |  |
| pSSI, n (%) | 5 (83.3) | 13 (72.2) | 1.00 |
| Anterior lesion, n (%) | 3 (50.0) | 16 (88.9) | 0.078 |
| Initial NIHSS (point), median (IQR) | 1.0 (0.0, 1.5) | 3.0 (2.0, 5.5) | 0.007^*^ |
| Worsened NIHSS (point), median (IQR) | 2.5 (1.8, 4.3) | 4.5 (3.0, 7.0) | 0.047^*^ |
| Severest NIHSS (point), mean ± SD | 3.8±2.2 | 9.3±3.8 | 0.003^*^ |

Abbreviations: SSI-ND indicates patients with single subcortical infarction with neurologic deterioration; PAD, parent artery disease; BMI, body mass index; SBP, systolic blood pressure; DBP, diastolic blood pressure; TG, triglyceride; TC, total cholesterol; HDL-C, high-density lipoprotein-cholesterol; LDL-C, low-density lipoprotein cholesterol; HbA1c, glycosylated hemoglobin; WBC, white blood cell count; NLR, neutrophil to lymphocyte ratio; Hs-CRP, high-sensitivity C-reactive protein; END, early neurological deterioration; pSSI, proximal single subcortical infarction; NIHSS, National Institute of Health Stroke Scale.

* *p* < 0.05 was considered statistically significant.
